# Review of the Progress of Medical and Surgical Electricity

**Published:** 1884-07-20

**Authors:** William R. D. Blackwood

**Affiliations:** Electrician and Neurologist to the Presbyterian Hospital, Physician to St. Mary’s Hospital, etc.


### REVIEW OF THE PROGRESS OF MEDICAL AND SURGICAL ELECTRICITY.

By William R. D. Blackwood, M.D.,
**Electrician and Neurologist to the Presbyterian Hospital, Physician to St. Mary's Hospital, etc.**

Galvanism has been successfully used in destroying the Dracunculus, or Guinea-worm, a terrible African pest. The worms, if broken off in extracting them, produce great irritation and sloughing, death resulting not unfrequently; but several cases are reported in the British Medical Journal, in which, galvanism being applied during traction on the worm, it came away entire.

Cutaneous faradization is recommended by Rumpf in progressive locomotor ataxia where the initial symptom is atrophy of the optic nerve; but the writer has never had good effects from faradism in ataxia of any kind, so far as durable results are concerned.

Thompson, of Indianapolis, reports in *The Archives of Ophthalmology*, vol. xii, p. 183, an interesting case of cystic orbital tumor treated by electrolysis. The tumor protruded downward and outward from tendo oculi behind the upper eyelid, and the motion of the eye was very limited; vision, $\frac{20}{0}$. A trocar and canula being introduced, ʒ iij of dark fluid were removed. The positive was connected with the canula, the negative applied over the temple of the same side, and a stabile current from a Stohrer of twenty cells passed for several minutes. Considerable inflammation occurred, and persisted for several days, but the cyst was destroyed, and in a month vision had risen to twenty thirtieths.

Neftel has returned to electrolysis in treating malignant tumors, destroying them at a single operation. A platinum anode is plunged perpendicularly into the tumor down to its presumed point of implantation, and from three to five cathodes placed on the periphery of the tumor. The current is then closed and rapidly carried to its greatest power (thirty to sixty elements). The position of the cathodes is changed about every five minutes, so as to cover every part of the tumor. The operation lasts about an hour. The tumor becomes livid, gray and, finally, black. There is a very slight general and local reaction. In two or three days the part operated upon becomes cold, and, after some discharge, finally comes away *en bloc*, leaving a denuded surface, which is soon covered by healthy granulations. Neftel has also treated benign tumors by this method, though they do not require such energetic treatment as those of the malignant type. The conclusions which he draws are:

1. Electrolysis is an antiseptic method, and as such may be combined with the ordinary methods of operation.

2. It is preferable to any other method in the treatment of malignant tumors.

3. Malignant tumors should be entirely destroyed by the operation, and at a single seance. In benign tumors it is sufficient to establish a retrograde metamorphosis.— *Virchow's Archiv.*

GALVANIZATION OF THE BRAIN, AND ITS VALUE IN THE TREATMENT OF CHOREA.—Charles L. Dana, M.D., details the effects of passing a galvanic current through the healthy human brain, the manner in which these effects are brought about, and gives the notes of eight cases of chorea, occurring in patients ranging in age from eight to fifteen years, treated more or less systematically by cerebral galvanization.

The average duration of the treatment was twenty-five days, of the symptoms thirty-four days, against six and eight weeks in thirty other cases, treated chiefly by arsenic. Dr. Dana, however, does not base his belief in the efficacy of electricity so much upon the statistics of duration as upon the fact—which occurred too frequently for mere coincidence—that evident improvement in the symptoms followed each seance, and continued for twenty-four or thirty-six hours.

What is claimed for "anodal galvanization" is that it is a most valuable adjunct in the treatment of chorea; that, if employed daily for a week or ten days, either with or without the simultaneous administration of arsenic, it materially shortens the duration of the majority of attacks occurring in children.

It is to be applied in the following way. A large sponge electrode of flexible brass, two by four inches, is thoroughly moistened with salt water. The hair of the patient is also thoroughly wetted, and the electrode applied over the side of the head above the ear. In hemi-chorea it need only be applied over the side opposite to the one affected. The other electrode is placed in the hand of the affected side. The electrode upon the scalp is made positive, and a stabile current of from three to six Stohrer's, or four to eight Daniell's cells, is passed from three to six minutes.

IN BASEDOW'S DISEASE.—Dr. Chvostek recommends (Centralbl. fur Klin. Med.) the following method:

1. The ascending constant current applied to the cervical sympathetic on each side for, at the most, one minute.

2. The same to the spinal cord (the anode at about the fifth dorsal spine, the cathode high up in the cervical region).

3. Through the occiput (one pole at each mastoid process), and in certain cases also through the temples, a constant current for, at the longest, one minute, and so weak that the patient can feel but the slightest sensation of burning. Sometimes, also, local galvanization of the thyroid gland with a weak constant current for about four minutes, the current to be reversed at the end of each minute. The application should be made every day.

ELECTRICITY AS A GALACTAGOGUE.—M. E. Labbee (*Union Medicale*) calls attention to the value of electricity as an adjuvant to other means for establishing or restoring the secretion of milk in puerperal women. When the secretion is absent or scanty, or when it has been suppressed from any cause, in addition to other modes of treatment (suction of the nipple, poultices of leaves of the Ricinus communis, etc.), it is well worth while to employ a weak current of electricity. The mammary gland is to be gently compressed between two electrodes consisting of moistened sponges. A mild current passed twice a day, for from ten to fifteen minutes at a time, will materially increase the functional activity of the gland. [The efficacy of static electricity in this direction has been reported in the Times by the writer, and confirmed by further observation in many cases.]

ELECTRICITY IN AURAL PRACTICE.—In nervous weakness, McBride, in "Clinical Notes on Ear-Disease," Archives of Otology, vol. xii, Nos. 3 and 4, reports success in several severe cases from induction-currents applied to either tragus, without any other treatment.

Dr. H. L. Morse reports his observation of cases of tinnitus aurium treated in Vienna. Galvanism was employed—the anode to the tragus, the cathode to the neck or other indifferent point. Weak currents were used in beginning, and these were gradually increased until sharp, stabbing sensations were experienced, the force being thereupon reduced slowly to zero. The dull, aching pain associated with many such cases was generally relieved promptly; often it was radically cured. If galvanism failed, faradic treatment usually was successful.

Electricity has also been extolled by Urbantschitsch in otalgia as valuable in relieving pain generally, without reference to the ultimate cause.

In the common earache of children, the writer has found galvanism successful after failure of chloroform-vapor, atropia and the ordinary methods employed.—*Med. Times.*

